# Microbial sulfate reduction by *Desulfovibrio* is an important source of hydrogen sulfide from a large swine finishing facility

**DOI:** 10.1038/s41598-021-90256-w

**Published:** 2021-05-21

**Authors:** Olga V. Karnachuk, Igor I. Rusanov, Inna A. Panova, Mikhail A. Grigoriev, Viacheslav S. Zyusman, Elena A. Latygolets, Maksat K. Kadyrbaev, Eugeny V. Gruzdev, Alexey V. Beletsky, Andrey V. Mardanov, Nikolai V. Pimenov, Nikolai V. Ravin

**Affiliations:** 1 Laboratory of Biochemistry and Molecular Biology, Tomsk State University, Tomsk, Russia 634050; 2 Institute of Microbiology, Research Center of Biotechnology, Russian Academy of Sciences, Moscow, Russia 119071; 3 Institute of Bioengineering, Research Center of Biotechnology, Russian Academy of Sciences, Moscow, Russia 119071

**Keywords:** Bacteria, Microbial communities, Environmental microbiology

## Abstract

There is still a lack of understanding of H_2_S formation in agricultural waste, which leads to poor odour prevention and control. Microbial sulfate reduction is a major process contributing to sulfide formation in natural and technogenic environments with high sulfate and low oxygen concentration. Agricultural waste can be considered a low-sulfate system with no obvious input of oxidised sulfur compounds. The purpose of this study was to characterise a microbial community participating in H_2_S production and estimate the microbial sulfate reduction rate (SRR) in manure slurry from a large-scale swine finishing facility in Western Siberia. In a series of manure slurry microcosms, we identified bacterial consortia by 16S rRNA gene profiling and metagenomic analysis and revealed that sulfate-reducing *Desulfovibrio* were key players responsible for H_2_S production. The SRR measured with radioactive sulfate in manure slurry was high and comprised 7.25 nmol S cm^−3^ day^−1^. Gypsum may be used as a solid-phase electron acceptor for sulfate reduction. Another plausible source of sulfate is a swine diet, which often contains supplements in the form of sulfates, including lysine sulfate. Low-sulfur diet, manure treatment with iron salts, and avoiding gypsum bedding are possible ways to mitigate H_2_S emissions from swine manure.

## Introduction

Swine yield is considered a dominant portion of the market available livestock trade^1^. Pork global production is forecasted to reach 102,160 thousand tons in 2021^2^, including 3.6 million tons in Russia, which is among the five top world producers along with China, the USA, EU, and Brasil. The acrid odour of swine manure is a well-known problem associated with livestock production facilities and animal waste storage systems^3^. The most noticeable and highly toxic odorous gas in animal wastes is hydrogen sulfide, which is recognised by its typical rotten-eggs smell. H_2_S and other volatile sulfur compounds comprise about one-half of the offensive odourants from swine manure^4,5^. Hydrogen sulfide has a low odour threshold of around 0.5 ppb (mg L^−1^) and causes eye irritation at concentrations of 50–100 ppm, while 300–500 ppm (mg L^−1^) may result in severe poisoning via inhibition of cytochrome oxidase in mammals^6^. Prolonged exposure to low concentrations of H_2_S was associated with persistent neurobehavioral dysfunction^7^. Odorous compounds also may affect the comfort, health, and production efficiency of animals as well as the health and comfort of human workers. Fatal asphyxia incidents were reported during manure handling/maintenance due to H_2_S poisoning^8^.

There was much uncertainty on the estimation of H_2_S emissions from livestock waste until recently. Proton-transfer-reaction mass spectrometry (PTR-MS) provided solid evidence that hydrogen sulfide from agricultural sources was a major source of atmospheric sulfur in regions with intensive animal production^9^. In contrast to the general perception of the minor importance of H_2_S compared to SO_2_ from industry, the authors demonstrated that emissions from finisher pig production comprised the largest source of atmospheric sulfur in Denmark. Emissions of H_2_S contribute to the atmospheric sulfur compounds via its oxidation to aerosol sulfate.

Sulfate-reducing prokaryotes have long been recognized to be major players in biogenic H_2_S production. Microbial sulfate reduction is ubiquitous in natural environments and mainly associated with marine biotopes due to their high sulfate level. Agricultural waste is considered as low-sulfate environment rich in organic compounds^4^. A highly active but cryptic sulfur cycle has been described for low-sulfate environments, such as peatlands^10^ and fresh-water lakes^11^. In low-sulfate biotopes, reduced sulfur species are rapidly reoxidised by oxygen- or iron-respiring microorganisms that sustain sulfate reduction rates as high as in sulfate-rich marine surface sediments^10^. Surprisingly, few reports are available on the presence and activity of sulfate-reducing bacteria (SRB) in swine manure. *Desulfovibrio*,* Desulfobulbus*, and *Desulfobacterium* have been detected by cloning of fragments of *dsrA* gene encoding a subunit of dissimilatory sulfite reductase from swine slurry stored in underground pits or lagoons^12^. Manure treatment with borax (sodium tetraborate decahydrate)^13^ and tannins^14^ were proposed to control SRB population and reduce H_2_S production. ZnO nanoparticles were applied as an additive to swine manure to reduce biogenic H_2_S emissions^15^. The *Desulfovibrio* was not detected in the microbial community from pig slurry was small, and that led authors to the conclusion that *Firmicutes* and *Bacteroidetes*, comprising the majority of microbial community, played an important role in the offensive odour compounds production via protein and carbohydrate degradations^16^.

The present study aimed to understand the diversity and activity of SRB in swine wastes produced by a large-scales swine finishing facility ‘Tomskii’ with a capacity of 176,000 hogs a year and located in the close vicinity of Tomsk, the capital city of the Tomsk region in Western Siberia, Russia. Strong odour from the facility reaches residential areas of the city in the summer time and is a matter of serious public concern. We identified bacterial consortia by 16S rRNA gene profiling and metagenomic analysis, measured sulfate reduction rate with radioactive tracer in manure slurry, isolated and studied SRB responsible for H_2_S production.

## Results

### Physicochemical characteristics of the sites

**Figure 1:**
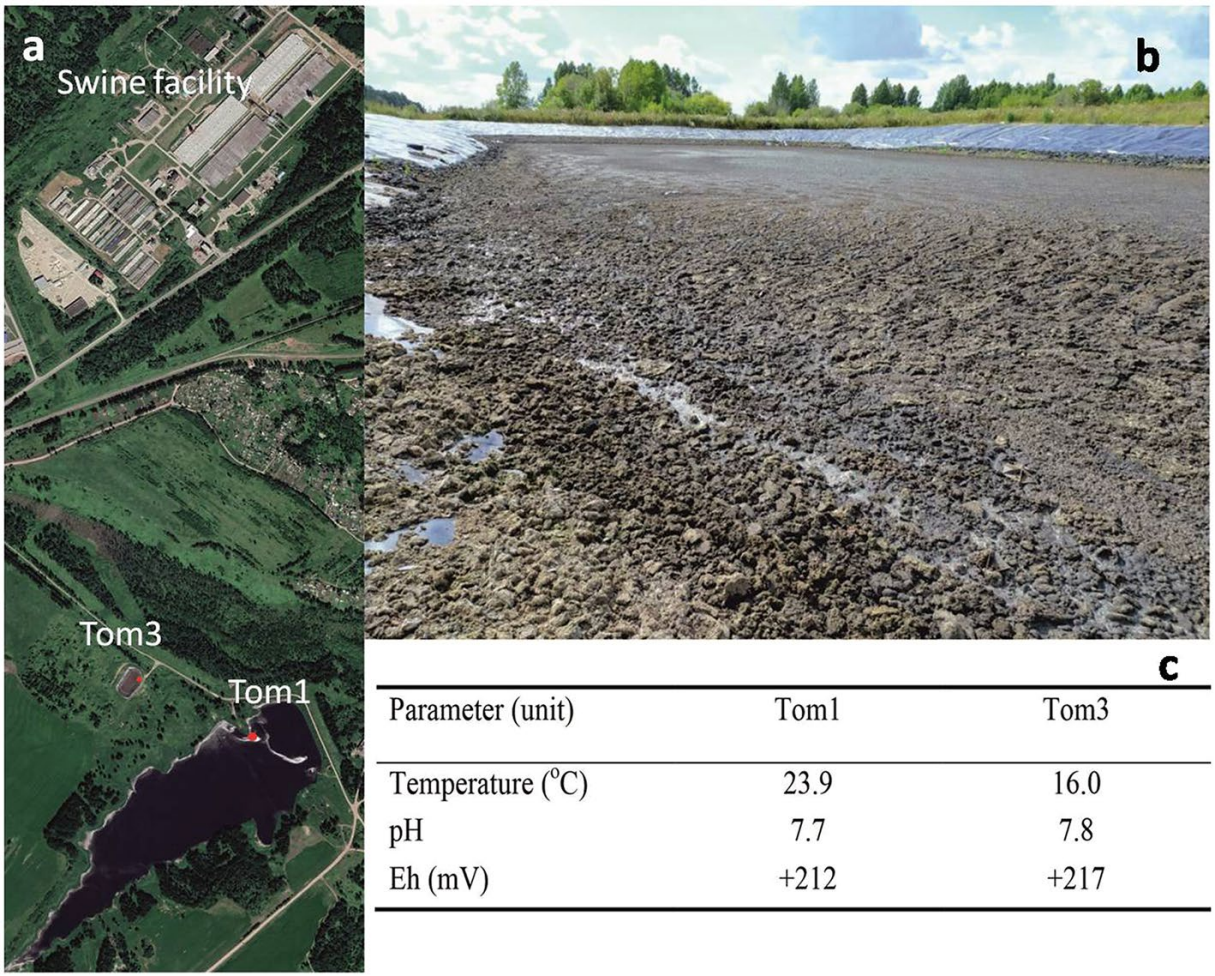
Google image of swine facility, manure storage lagoon, and solid–liquid separation lagoon with sampling locations (**a**), manure lagoon (**b**), and characteristics of the sites at the time of sampling (**c**). Maps Data: Google, CNES/Airbus, Landsat/Copernicus, Maxar Technologies, 2021.

Waste samples were collected from two lagoons at the large-scale swine finishing facility ‘Tomskii’. The swine manure and wastewater from the facility is treated by the lagoons only. The cumulative manure slurry from the facility pumped to a manure storage lagoon (N56° 58′, E85° 14′; Tom3), which connected by a pipe system with a larger solid–liquid separation lagoon (N56° 57′, E85° 14′; Tom1) of a total volume around 1.5 billion m^3^ (Fig. 1). The solid–liquid separation lagoon water was used to fertilise agricultural fields via a sprinkler system. However, this practice was recently abandoned due to the public complaints of malodour from the site. Part of the solid–liquid separation lagoon effluent is released into small stream by an underdrain.

**Figure 2:**
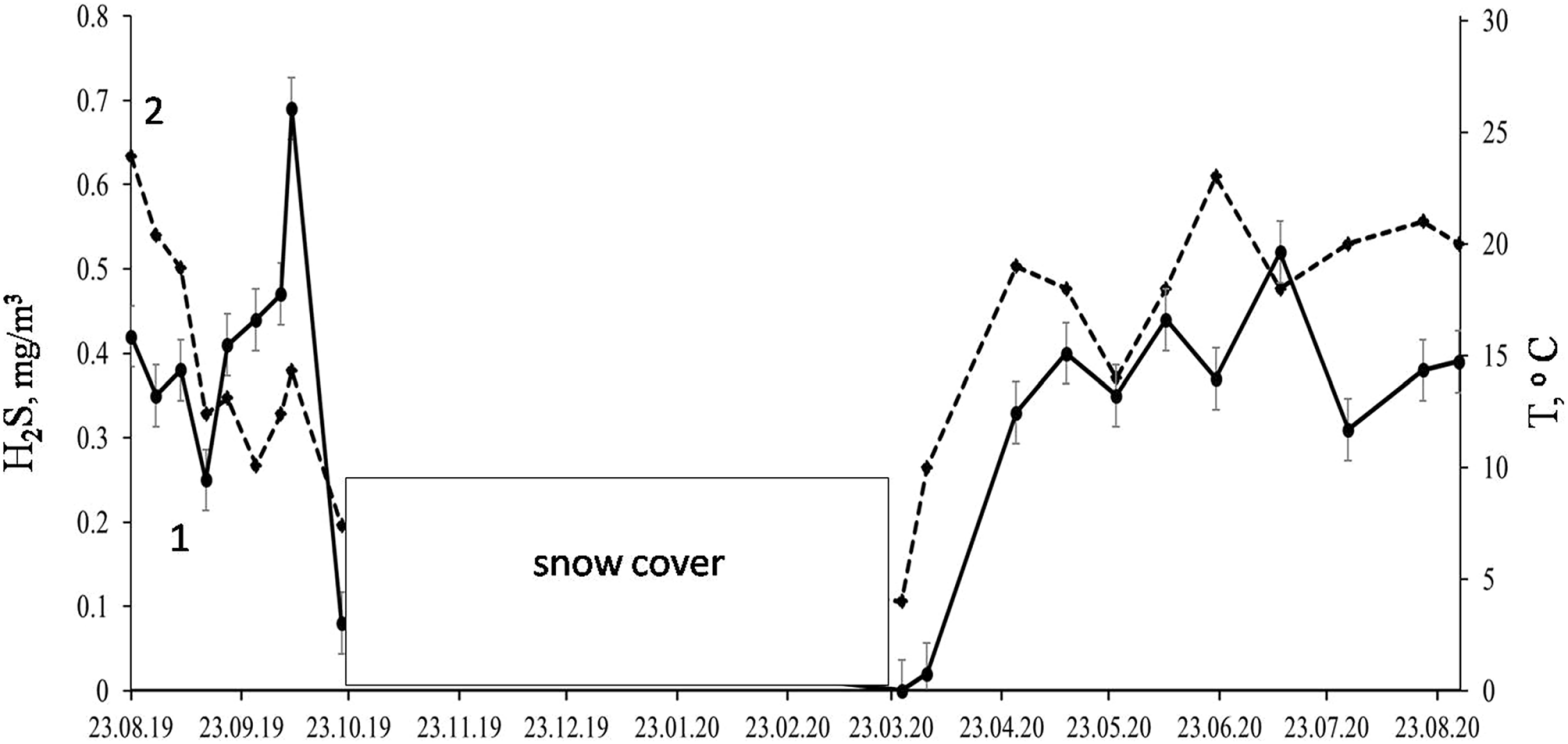
Time course of hydrogen sulfide levels (1, solid line) and ambient air temperature (2, dashed line) at the solid–liquid separation lagoon (Tom1). H_2_S concentrations in air were measured via an OKA-T portable gas analyser with electrochemical sensor (accuracy of ± 25% of reading). Vertical bars show the standard deviation calculated from three readings.

**Figure 3:**
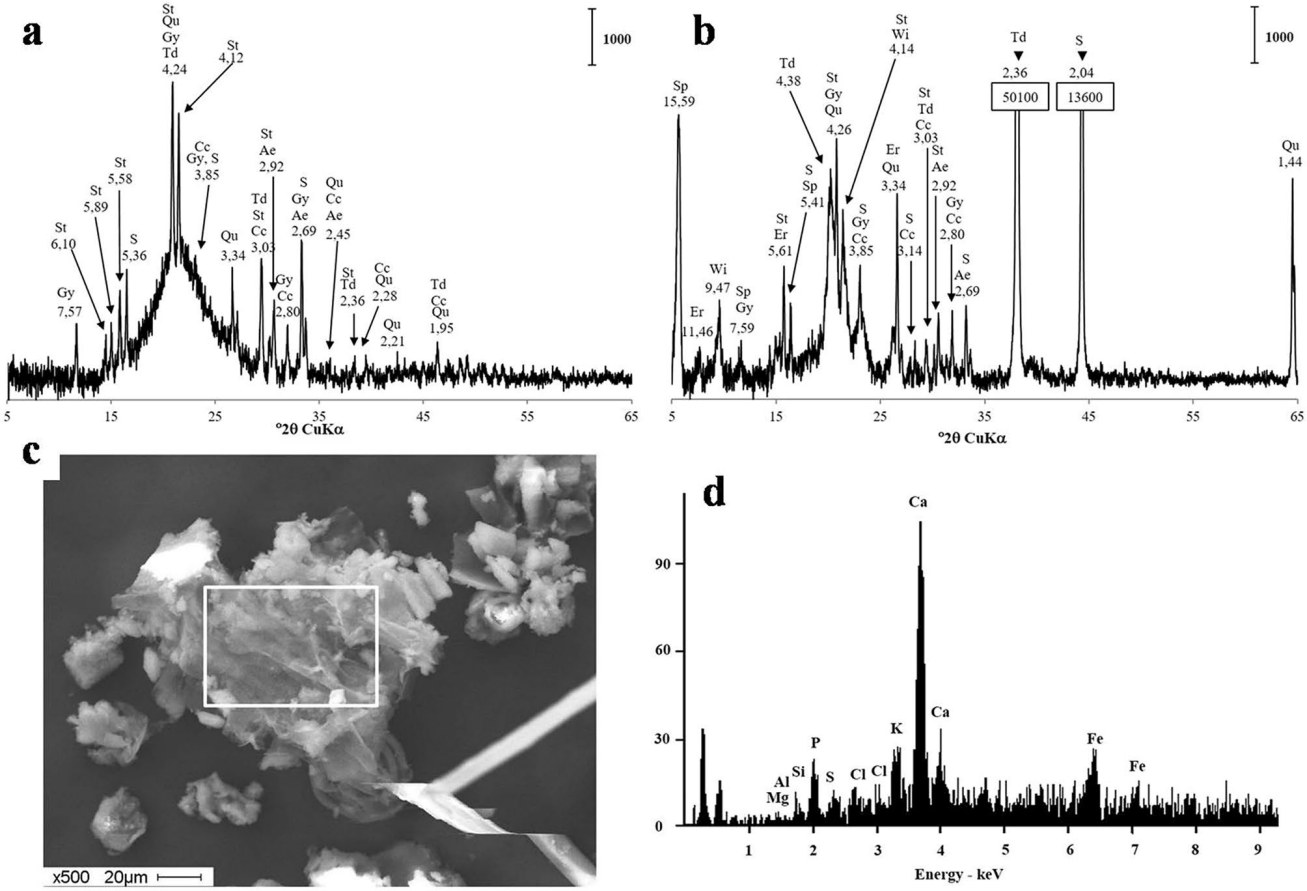
The X-ray diffractograms show the mineralogical composition of the sediment from the solid–liquid separation lagoon, sample Tom1 (**a**) and manure slurry, sample Tom3 (**b**). The vertical bar shows the scale of relative counts. Letter codes: *Ae* ankerite, Ca(Fe,Mg,Mn)(CO_3_)_2_, *Cc* calcite, CaCO_3_, *Er* Erionite, (Na_2_,K_2_,Ca)_2_Al_4_Si_14_O_36_·15H_2_O, *Gy* gypsum,CaSO_4_·2H_2_O, *Qu* quartz, SiO_2_, *S* sulfur, S_6_, *Sp* Saponite 15ACa_0.2_Mg_3_(Si,Al)_4_O_10_ (OH)_2_·4(H_2_O), *St* Struvite, NH_4_MgPO_4_·6H_2_O, *Td* tridymite, SiO_2_, Wi willhendersonite, KCaAl_3_Si_3_O_12_·5H_2_O. Note gypsum and sulfur occurrence in both samples. SEM micrograph (**c**) and the respective microprobe analysis (**d**) demonstrates elemental composition of the manure slurry, sample Tom3.

The H_2_S concentration in the ambient air at the solid–liquid separation lagoon (Tom1) was monitored over time (Fig. 2). No measurements were taken during the seasonal snow cover, which lasted from the beginning of November till the end of March. The H_2_S concentration in ambient air changed from 0.08 (late October) to 0.69 mg m^−3^ (mid-September). These concentrations well exceed the maximum admissible level of 0.008 mg m^−3^ set up in Russia for residential areas. The average ambient air H_2_S level tended to correlate with outdoor temperature. The pH values of both lagoons were 7.7–7.8 and redox potential of + 212–217 mV, indicative of aerobic conditions (Fig. 1). The sulfate concentration in the pore water was 17.4 mg L^−1^, and chloride was the major anion with the concentration of 300 mg L^−1^ (Table 1). Dissolved iron was low and did not exceed 1.36 mg L^−1^.The mineralogical composition of the sediment and manure slurry revealed the presence of gypsum (CaSO_4_·2H_2_O) (Fig. 3). The manure also contained crystalline sulfur (PDF-24-0733). Calcium carbonates, calcite, CaCO_3_ (PDF-24-0027), and ankerite (Ca(Fe,Mg,Mn)(CO_3_)_2_; PDF-79-1347) were also present in both samples. Scanning electron microscopy with X-ray microanalysis (SEM–EDS) showed that Ca reached up to 51% of the elemental composition of solids and S up to 14%. Fe and P comprised up to 20% and 54% of the elemental composition in the solids, respectively.

**Table 1:** Physical and chemical characteristics of the pore water from the solid–liquid separation lagoon (Tom1) and manure storage lagoon (Tom3).

Parameter (unit)	Tom1	Tom3
Temperature (°C)	23.9	14.3
pH	7.7	7.8
Eh (mV)	+ 212	+ 217
Na (mg L^−1^)	176	254
B (mg L^−1^)	0.22	0.32
K (mg L^−1^)	470	655
Ca (mg L^−1^)	126	177
Mg (mg L^−1^)	30.7	63.1
As (mg L^−1^)	0.054	0.0074
Se (mg L^−1^)	0.013	0.015
Rb (mg L^−1^)	0.32	0.63
Sr (mg L^−1^)	0.54	0.88
Ba (mg L^−1^)	0.04	0.100
Si (mg L^−1^)	32.2	35.79
Fe (mg L^−1^)	1.07	1.36
Co (mg L^−1^)	0.0061	0.0026
Ni (mg L^−1^)	0.34	0.063
Cu (mg L^−1^)	0.121	0.16
Zn (mg L^−1^)	0.11	0.36
Cl^−^ (mg L^−1^)	ND	300
PO_4_^−^ (mg L^−1^)	ND	27.4
NO_3_^−^ (mg L^−1^)	ND	1.5
SO_4_^2−^ (mg L^−1^)	ND	17.4

*ND* not determined.

### Sulfate reduction rate

**Figure 4:**
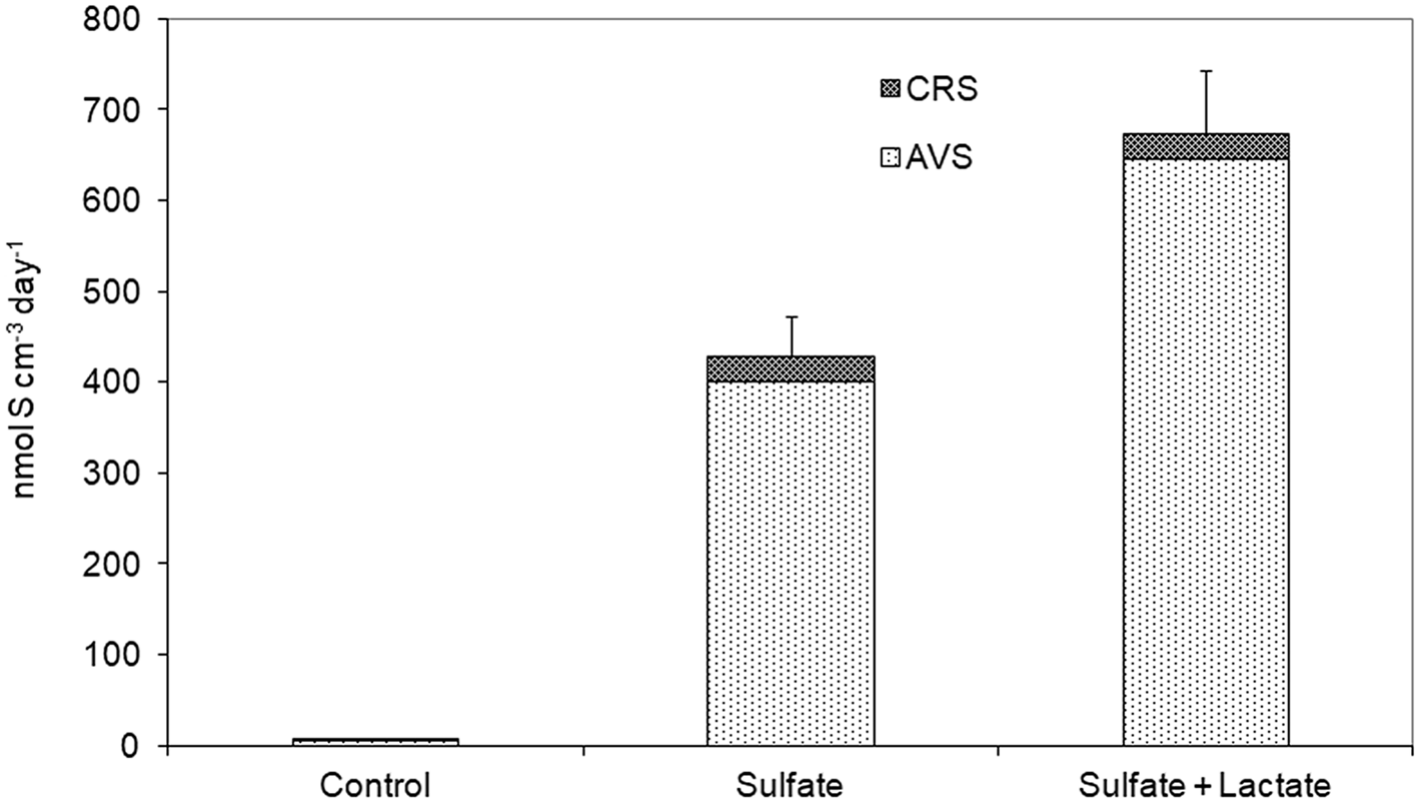
Sulfate reduction rate (SRR) and the amount of sulfur in acid volatile sulfides (AVS) and chromium-reducible sulfur (CRS) measured directly in the manure slurry (Tom3) without amendments (control), manure slurry supplemented with sulfate (Sulfate), and manure slurry supplemented with sulfate plus lactate (sulfate + lactate).

The sulfate reduction rate (SRR) measured directly in manure slurry (sample Tom3) was high and reached 7.25 nmol S cm^−3^ day^−1^. The acid volatile sulfide fraction (AVS), which includes H_2_S and FeS, was the major product of ^35^SO_4_^2−^ reduction, and comprised 69% of total reduced sulfur. Sulfate amendment enhanced the rate up to 427 nmol S cm^−3^ day^−1^ (Fig. 4). Lactate plus sulfate had the most pronounced effect on SRR and increased it by nearly 100 times over the control to 674 nmol S cm^−3^ day^−1^. The experiments with amendments showed that the higher SRR, the smaller is the fraction of pyrite sulfur (CRS) in the total pool of reduced sulfur.

### Microbial community structure recovered by 16S rRNA gene profiling

The preliminary investigation of the microbial community by 16S rRNA sequencing did not reveal any taxonomic groups with known capability for dissimilatory sulfate reduction in the sediment slurry from solid–liquid separation lagoon (Tom1). Considering the low sulfate concentration in the biotope, we set up a microcosm series of the sediment slurry from solid–liquid separation lagoon (Tom1) amended with sulfate, and sulfate plus lactate with the purpose to reveal low-number SRB limited by electron donor or/and acceptor. The same amendments were applied to the manure lagoon slurry microcosms (Tom3). All microcosms were supplemented by control without amendments. The H_2_S content in microcosms was monitored and reached 16 ± 2 mg/L in Tom1 and 19 ± 4 mg/L in Tom3 microcosms. Microbial communities of the sediment slurry from solid–liquid separation lagoon (Tom1), manure slurry from the manure lagoon (Tom3), and corresponding microcosms were characterised by analysing their 16S rRNA gene sequences. At least 11,000 16S rRNA gene reads (on average 33,316 for Tom1 and 25,760 reads for Tom3 samples) were used to reveal microbial community composition.

**Figure 5:**
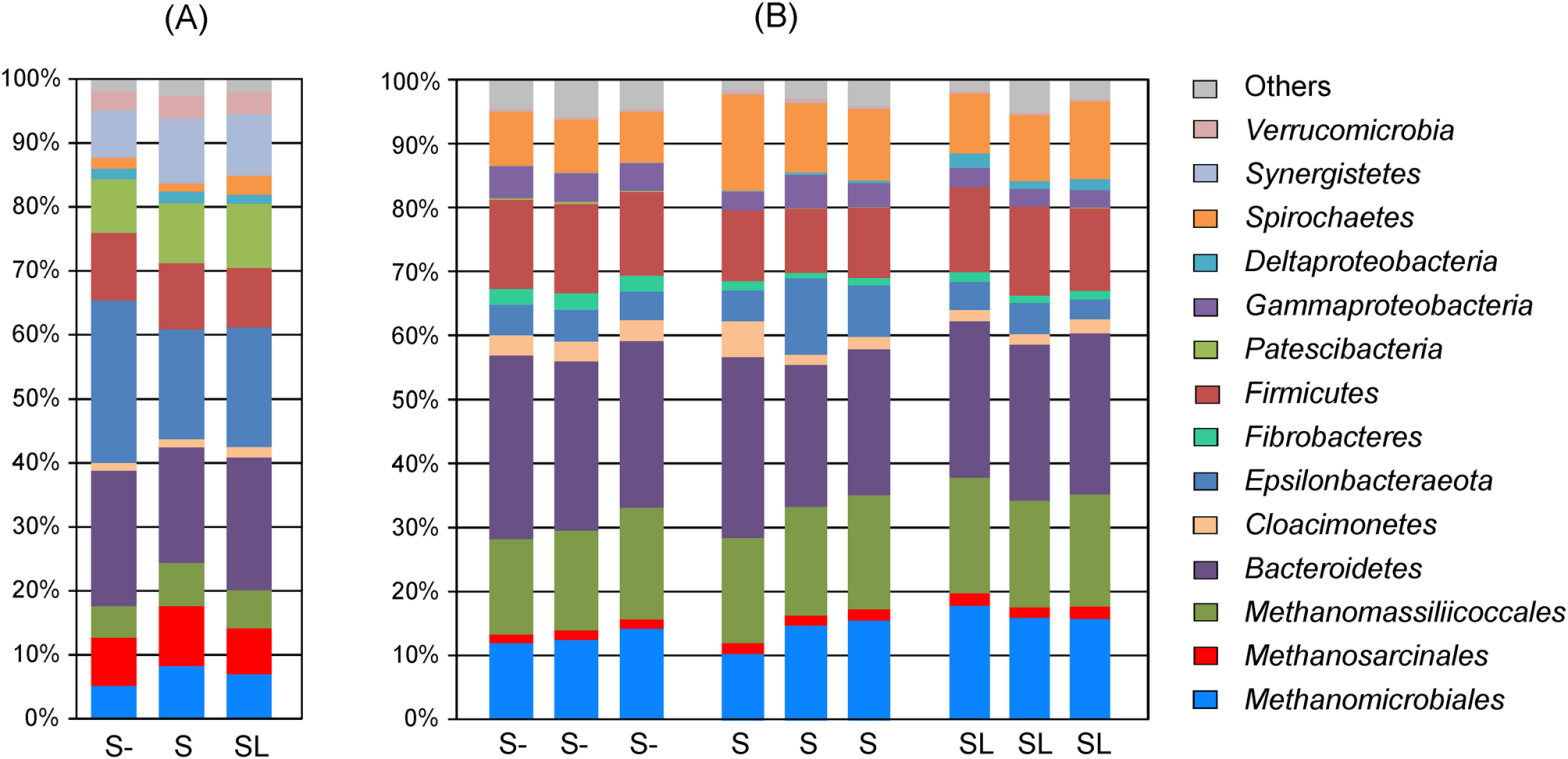
The relative abundance of taxonomic groups of microorganisms according to 16S rRNA gene profiling in microcosms derived from (**A**) the sediment slurry (Tom1) and (**B**) manure slurry (Tom3). S-, control microcosms with no supplements; S and SL, microcosms amended with sulfate, and sulfate + lactate, respectively. Note that manure slurry samples were analysed in triplicate.

Sulfate and lactate amendments had little effect on the composition of the microbial community of the sediment slurry from solid–liquid separation lagoon (Tom1), and all three samples were dominated by methanogenic archaea (18.3% to 25.4% of total 16S rRNA gene reads), members of the phyla *Bacteroidetes* (18.1–21.1%), *Campylobacteraeota* (17.2–25.3%), *Firmicutes* (9.4–10.6%), *Patescibacteria* (8.4–10.0%), and *Synergistetes* (7.4–10.2%) (Fig. 5A). *Deltaproteobacteria* accounted for 1.4–1.8% and were represented by *Smithella* sp., syntrophic degraders of low molecular weight organics^17^. Lineages with known capability for dissimilatory sulfate reduction were not found in any of the Tom1 microcosms. The only detected lineage known to be involved in the sulfur cycle, bacteria of the genus *Sulfurimonas* (Campylobacterota), were present below the detection limit in the control microcosm without sulfate and accounted for 0.9% and 5.3% in microcosms amended with sulfate and sulfate with lactate, respectively. *Sulfurimonas* species are typically chemolithoautotrophs found in sulfidic environments, such as hydrothermal vents, marine sediments, sulfidic springs, and groundwater^18^. They can oxidise reduced sulfur compounds and hydrogen using oxygen, nitrate, or nitrite as electron acceptors.

Most of dominant prokaryotic lineages in the sediment slurry samples from the manure lagoon (Tom1) were also abundant in the manure slurry microcosms (Tom3), namely methanogenic *Euryarchaeota* (29.6–38.2%), *Bacteroidetes* (22.0–28.5%), *Campylobacterota* (3.1–11.9%), and *Firmicutes* (10.0–13.9%) (Fig. 5B). *Spirochaetes* (8.0–15.0%) and *Proteobacteria* (3.0–5.6%) were also abundant in Tom3, while *Patescibacteria* and *Synergistetes* accounted for less than 0.5% of the 16S rRNA gene reads in all Tom3 samples. Several presumably sulfate-reducing bacterial lineages were identified in the manure slurry microcosms (Tom3): *Deltaprotreobacteria* of the families *Desulfovibrionaceae*, *Desulfomicrobiaceae*, *Desulfobacteraceae*, and *Desulfobulbaceae*. All these groups except *Desulfovibrionaceae* were present in minor amounts (< 0.1% in all Tom3 samples), and their relative abundance was not impacted by the addition of sulfate. On the contrary, *Desulfovibrionaceae* accounted for 0.07%, on average, of the control Tom3 community. Their abundance increased to 0.28% in the sulfate-amended microcosm and up to 1.72% in the microcosm supplemented with sulfate and lactate. Nearly all *Desulfovibrionaceae* 16S rRNA gene sequences were assigned to the genus *Desulfovibrio*. *Sulfurimonas* sp. were found only in minor amounts (< 0.1%), and their relative abundance did not significantly differ between the Tom3 samples.

### Metagenomic analysis revealed sulfate-reducing bacteria in the manure sample

To reveal sulfate-reducing microorganisms in the swine manure, we sequenced the metagenome of the manure sample from the manure lagoon (Tom3) used to inoculate the microcosms and searched for genes *dsrA*, *dsrB* and *dsrD*, key markers for the dissimilatory sulfate reduction pathway^19–21^. Of about two million metagenomics reads, 188, 156 and 24 were mapped to the *dsrA*,* dsrB* and *dsrD* datasets, respectively (Table 2). Most reads (72–81%) were taxonomically assigned to the family *Desulfovibrionaceae*, primary to *Desulfovibrio* sp. Considering the *Deltaproteobacteria*, *dsr* sequences most closely related to ones from members of the families *Desulfobacteraceae*, *Desulfobulbaceae*, *Desulfomicrobiaceae*, and *Syntrophaceae* were found in minor numbers (Table 2). Sulfate-reducing lineages of *Firmicutes*, members of the families *Peptococcaceae* and *Syntrophomonadaceae*, comprised less than 10% of all *dsr* reads. A few *dsrA* and *dsrB* reads were assigned to the *Betaproteobacetria* and probably represented the reversely operating dissimilatory sulfite reductase, involved in sulfur oxidation^22^.

**Table 2:** Taxonomic assignment of *dsr* reads.

Lineage	*dsrA*		*dsrB*		*dsrD*	
	Reads	% of the total	Reads	% of the total	Reads	% of the total
***Deltaproteobacteria***						
*Desulfobacteraceae*	3	1.6	6	3.8	4	16.7
*Desulfobulbaceae*	8	4.3	13	8.3	2	8.3
*Desulfomicrobiaceae*	6	3.2	5	3.2	0	0.0
*Desulfovibrionaceae*	153	81.4	113	72.4	18	75.0
*Syntrophaceae*	0	0.0	2	1.3	0	0.0
***Firmicutes***						
*Peptococcaceae*	11	5.9	12	7.7	0	0.0
*Syntrophomonadaceae*	3	1.6	2	1.3	0	0.0
***Betaproteobacteria***	1	0.5	3	1.9	0	0.0
**Unclassified**	3	1.6	0	0.0	0	0.0
Total	188	100	156	100	24	100

### Targeted *Desulfovibrio* isolation from Tom1

In accordance with our 16S rRNA profiles of manure slurry, the attempts to isolate *Desulfovibrio*, the major drivers of sulfate reduction, were made. *Desulfovibrio* are considered important hydrogen consumers and can also grow on formate, lactate, pyruvate, and many other simple organic compounds. Formate was chosen as an electron donor to prevent overgrowth by other heterotrophic microorganisms. A set of enrichments with 7.5 mM formate supplemented with zero-valent iron was set up. The vials were inoculated directly from the Tom1sediment slurry. The additional discrimination against non-sulfate-reducing microorganisms was made using a solid medium, which allowed for isolating black colonies indicative of sulfide production. Cultures originating from the colonies were purified by serial dilutions in liquid Widdel-Bak (WB) medium. This approach resulted in fast isolation of two pure cultures, designated L2 and L4. The 16S rRNA genes of the isolates were amplified and sequenced, demonstrating the strain L4 belonged to *Desulfovibrio desulfuricans* and showed 99.94% sequence similarity with the type strain *D. desulfuricans* Essex 6. The average nucleotide sequence identity (ANI) between the genomes of L4 and Essex 6 was 98.62%, a value above the species boundary cutoff of 95%^23^, indicating that L4 is a new strain of *Desulfovibrio desulfuricans.* The closest relative of strain L2 is another model SRB, *Desulfovibrio vulgaris* Hildenborough, with the sequence similarity of 99.78%.

The genome of strain L4 consisted of a chromosome of 3,544,025 bp and a 10,876 bp long plasmid, designated pDsulf-L4. Analysis of *Desulfovibrio* L4 genome revealed the presence of complete set of genes for dissimilatory sulfate reduction, including sulfate transporter, sulfate adenylyl transferase, adenylsulfate reductase *aprAB* and dissimilatory sulfite reductase (*dsrABD* and *dsrC*). Genes for the adenylsulfate reductase-associated electron transfer complex QmoABC and sulfite reductase-associated electron transport proteins DsrMKJOP were also found. The plasmid was sequenced with 1885-fold average coverage, a value 6.2 times higher than the L4 chromosome. Interestingly, highly similar plasmids (> 99.4% nucleotide sequence identity over the whole length of pDsulf-L4) were found not in *Desulfovibrio* sp., but in enterobacteria, namely in *Salmonella enterica* subsp. enterica serovar Manhattan strain SA20084699 (31,173-bp long plasmid), *Salmonella enterica* subsp. enterica serovar Muenchen strain CVM 20744 (plasmid p20744-1), and *Shigella flexneri* 1c strain Y394 (plasmid pNV-Y394). Plasmid pDsulf-L4 harboured genes *mobA* and *mobC* associated with plasmid transfer and replication genes *repA* and *repC*. Like pNV-Y394^24^, plasmid pDsulf-L4 contained a multidrug-resistance cassette consisting of tetracycline resistance gene *tetA* (MFS family exporter), streptomycin resistance genes *strA-strB* (aminoglycoside phosphotransferases), and sulfonamide-resistance dihydropteroate synthase gene *sul2*.

## Discussion

Our data demonstrate that SRB constitute a minor component of the swine manure microbial community, however their role in malodorous H_2_S production is immensely high. Sulfate reduction measured in manure slurry with a radioactive tracer reached a high rate of 7.25 nmol S cm^−3^ day^−1^ and was the same order of magnitude as SRR known for a model ecosystem, estuarine and shallow sea sediments, where sulfate reduction has shown to be most active and accounts for about 20–40% of the global sulfate reduction^25^. Several previous indirect observations on SRB activity in swine manure^12–14^ confirm our conclusion of the important role of SRB in H_2_S production. For instance, a direct correlation between the initial sulfate concentration in swine manure slurry and the amount of sulfide produced was reported^26^. A microcosm experiments revealed that sulfate reduction in the manure slurry was limited by sulfate concentration and availability of low weight electron donors for SRB, like lactate. O_2_-respiring aerobes likely outcompete SRB for small organic molecules, which serve as electron donors for respiration. The soluble sulfate in waste slurry may originate from swine diet, in which nutrients and supplements, such as lysine, copper, iron, and zinc are often formulated as sulfate salts. Previous research indicated that sulfate in slurry ultimately originates from the minerals or premix in the feed and, to some extent, from S compounds in drinking and wash water^4,26^, which implies that SRB activity can be controlled by lowering the dietary S level. Air bubbling and dietary S reduction were suggested as viable methods for reducing peak H_2_S emissions from swine manure slurry^4^. However, the methods have been applied at bench scale reactors and were not validated at larger scales.

The sulfate ion concentration measured in the manure slurry from the manure lagoon in our research comprised 17.4 mg/L, which is not sufficient to sustain a constant H_2_S flux observed in the ambient air. X-ray diffraction (XRD) analysis revealed gypsum occurrence in manure (Tom3) and sediment slurry (Tom1). CaSO_4_ may serve as a solid-phase electron acceptor for SRB. Sulfide formation by *Desulfovibrio* spp. from gypsum was shown to be almost compatible in rate and quantity to that produced from soluble sulfate^27^. Gypsum bedding is often used in animal production to improve animal welfare and provide agronomic benefits to manure recycled back to the land^28^. It was reported that the monitoring of ten farms revealed that the gypsum-containing manure storages produced H_2_S levels above recognised safe thresholds for both livestock and humans^29^. The use of the sulfate moiety from gypsum by SRB in the studied samples is indirectly confirmed by the observed presence of calcium carbonates, which are proved to be produced as a result of dissimilatory sulfate reduction^30^.

The small share of pyrite and the other chromium-reducible sulfur (CRS), revealed in our experiments with radioactive tracer, clearly indicates the iron deficiency in the manure slurry. Elemental analysis by ICP-MS detected low Fe concentration, which did not exceed 1.07 mg L^−1^ in Tom1 and 1.36 mg L^−1^ in Tom3 sample. The XRD analyses indicated the presence of crystalline elemental sulfur in the manure slurry sample. The H_2_S produced by SRB under low-Fe conditions is easily oxidised biologically and chemically to elemental sulfur. The latter is more reluctant to oxidative conditions measured in the manure slurry sample. The presence of elemental sulfur explains the occurrence of sulfur-oxidising *Sulfurimonas* revealed by 16S rRNA-profiling. Thus, sulfur-oxidisers may oxidise hydrogen sulfide and elemental sulfur back to sulfate and sustain the bacterial sulfate-reduction. The low-iron conditions of the swine waste point out to the possible solution to minimise sulfide emissions from the wastes by adding iron salts. The dosage of iron salts is a common and well-documented practice to reduce sulfide concentration in municipal sewage conveyance systems^6,31^. Ferrous iron (Fe^2+^) reacts with sulfide (S^2−^) and precipitates it in the form of ferrous sulfides with low solubility.

Our data suggest that *Desulfovibrio* are the major players in H_2_S production from swine manure. This finding corroborates with the observation that *Desulfovibrio* spp. were predominant SRB in piglet gut^32^. Recently, a new species *Desulfovibrio porci* isolated from pig faeces was described under a large cultivation project, called ‘Pig intestinal bacterial collection’^33^. *Desulfovibrio* are often found in human and animal gut^34^, where they can utilise hydrogen, produced by fermentation^20^. H_2_S has been suggested to play a potential role in the etiology of bowel disorders such as the inflammatory bowel diseases, particularly ulcerative colitis^35^. We have used formate for targeted isolation of the bacteria. Analysis of *Desulfovibrio* L4 genome revealed the presence of formate dehydrogenase and group 1b respiratory H_2_-uptake [NiFe] hydrogenase that could enable growth on formate and hydrogen coupled to sulfate reduction. In addition, oxygen-tolerant group 1d hydrogenase could operate under microaerophilic conditions (that could occur in the manure slurry), although SRB generally cannot compete with oxygen-respiring microorganisms for the low-weight organic electron donors, like lactate.

The occurrence of plasmids is not often reported in *Desulfovibrio*. *D. vulgaris* Hildenborough contains a large plasmid of 202 kb, which encodes for nitrogenase and catalase^36^. Strain L4 was found to harbor a medium-copy number plasmid pDsulf-L4, encoding replication and mobilization functions, as well as an antibiotic-resistance cassette, predicted to confer resistance against sulfonamide, streptomycin, and tetracycline. To the best of our knowledge, the mobile elements carrying antibiotic-resistance genes of clinical importance were not reported in *Desulfovibrio* sp. so far. Interestingly, nearly identical plasmids were found in several clinical isolates of *Enterobacteriaceae*, particularly in *Shigella flexneri* 1c strain Y394 (plasmid pNV-Y394)^24^. Therefore, horizontal acquisition of this plasmid by *Desulfovibrio* sp. L4 from the pig gut microbiome is a plausible scenario. Antibiotics, including tetracycline, streptomycin, and sulfonamides, are widely used for disease prevention in food animals in Russia, promoting selection of a resistant gut microbiome. Although pNV-Y394 lacks complete conjugative transfer elements and is non-conjugative^24^, it contained mobilization genes that could contribute to its transfer. The occurrence of *Desulfovibrio* sp. in pig intestines^33^ suggests that this transfer could have occurred already in the gut and was followed by dissemination of a drug-resistant *Desulfovibrio* strain in the environment.

Recent bioinformatic analysis based on metagenomes and metagenome-assembled genomes (MAGs) expended the capacity for sulfate/sulfite reduction to 13 bacterial and archaeal phyla for which the ability to dissimilatory reduction was previously unknown^21^. These findings imply that the swine manure analysed in our study might include representatives of novel sulfate-reducers participating in H_2_S production. However phylogenetic analysis based on 16S rRNA did not reveal any prokaryotes belonging to *Acidobacteria*, *Actinobacteria*, *Candidatus* Desantisbacteria, *Candidatus* Hydrothermarchaeota, *Candidatus* Schekmanbacteria, *Candidatus* Zixibacteria, *Chloroflexi*, and *Planctomycetes* for which the potential dissimilatory sulfate reduction pathway was identified. More important, in our experiments *Desulfovibrio* was the only phylogenetic group which share in the microbial community increased in the sulfate-amended microcosms. Thus, with a high probability *Desulfovibrio* were the key players in sulfate reduction and H_2_S production from swine manure.

## Methods

### Study site, sampling, and physicochemical parameters measurements

The near-bottom sediment slurry from the solid–liquid separation lagoon (site Tom1) was sampled on 12 July 2019. Manure slurry was sampled from the storage lagoon (site Tom3) on 18 October 2019. The pH, temperature, and redox potential measurements (Hanna Instruments, Vöhringen, Germany) were taken on-site by inserting the appropriate electrodes into the slurry. H_2_S concentrations in air were measured via an OKA-T portable gas analyser (Informanalitika, Russia) with electrochemical sensor (accuracy of ± 25% of reading). Anion concentrations in the pore water samples were measured using ion chromatography (Dionex). H_2_S was measured colourimetrically in triplicate with the methylene blue method^37^ using a Smart Spec Plus spectrophotometer (Bio-Rad Laboratories, Hercules, CA). The elemental composition of the manure storage lagoon and solid–liquid separation lagoon was analysed by ICP-MS (Plasma Chemical-Analytical Centre, Tomsk). The mineralogical composition of the Tom1 sediment and Tom3 manure slurry were characterised by X-ray diffraction (XRD) using a Shimadzu XRD-6000 diffractometer as previously described^38^. Air-dried sediment and slurry were examined under a Philips SEM 515 scanning electron microscope (SEM). Energy dispersive spectrometry (EDS) using an EDAX Inc. spectrometer (Mahwah, NJ) was performed at a voltage of 30 kV and a working distance of 12 mm.

### Measurement of sulfate-reduction rates

Radioactive sulfate was used to determine the sulfate-reduction rates (SRR). Manure slurry was placed in sterile 30 mL culture bottles without a headspace (sealed with butyl rubber stoppers), which received aliquots (200 µL) of Na_2_^35^SO_4_ (4 µCi ‘Perkin-Elmer’, USA) by injection through the butyl rubber stopper. The effect of a potential electron donor and acceptor on in situ sulfate reduction was examined with the following amendments: 18 mM lactate and 28 mM sulfate. The bottles were incubated in the dark at the in situ temperature for 24 h followed by addition of 1 mL of 2 M KOH to terminate the reaction and to fix sulfide. Radioactivity was measured in the acid volatile sulfide (AVS), H_2_S and FeS, and chromium-reducible sulfur (CRS) fractions, which included pyrite, and elemental and organic sulfur, as previously described^39,40^. The average SRR and standard deviation were calculated from triplicate incubations.

### Experiments with sulfate and lactate amendments

Experiments with sulfate and lactate amendments were run to test their effect on the composition of the microbial community. Serum bottles (500 mL) were used for the experiments, to which 300 mL of manure or sediment slurry and 200 mL of artificial medium were added. The medium had the following composition (per litre): 0.2 g KH_2_PO_4_, 0.25 g NH_4_Cl, 1 g NaCl, 0.4 g MgCl_2_·6H_2_O, 0.5 g KCl, and 0.113 g CaCl_2_. The experimental microcosms were exposed to sulfate and sulfate + lactate amendments. The SO_4_^2−^ treatment contained 28 mM sulfate in the form of Na_2_SO_4_ and the sulfate + lactate treatment received 18 mM lactate (as sodium salt) and 28 mM sulfate (as Na_2_SO_4_). For manure slurry samples (Tom3), each treatment as well as a control without supplements was run in triplicates. Anoxic replicates were sealed with butyl rubber stoppers and crimp-on aluminum seals allowing sampling with a syringe. The microcosms were incubated in darkness in a temperature-controlled room (20 ℃). The bottles with sediment slurry (Tom1) were incubated for 15 days. The microcosms with manure slurry (Tom3) were run until the pronounced increase of H_2_S in the bottle, which occurred after 30 h from the beginning of the experiment. For each microcosm incubation, the total DNA was extracted from 1 mL of the slurry using the DNeasy Power Max Soil Kit (Qiagen, Hilden, Germany).

### 16S rRNA–based microbial community profiling

PCR amplification of 16S rRNA gene fragments comprising the V3–V6 variable regions was carried out using the universal bacterial primers 341F (5′-CCTAYGGGDBGCWSCAG) and 806R (5′-GGACTACNVGGGTHTCTAAT). PCR fragments were bar coded using the Nextera XT Index Kit v.2 (Illumina, San Diego, CA, USA). The PCR fragments were purified using Agencourt AMPure beads (Beckman Coulter, Brea, CA, USA) and quantitated using the Qubit dsDNA HS Assay Kit (Invitrogen, Carlsbad, CA, USA). Then, all of the amplicons were pooled together in equimolar amounts and sequenced on the Illumina MiSeq (2 × 300 nt paired-end reads). Overlapping paired reads were merged using FLASH v.1.2.11^41^.

The data obtained for experiments with sediment slurry (Tom1) and with manure slurry (Tom3) were analysed separately. All sequences obtained in a given experiment (three samples for Tom1 or nine samples for Tom3) were clustered into operational taxonomic units (OTUs) at 97% identity using the USEARCH v. 11 program^42^. Low quality reads, chimeric sequences, and singletons were removed during clustering by the USEARCH algorithm. To calculate OTU abundances, all reads obtained for a given sample (including singleton and low-quality reads) were mapped to OTU sequences at a 97% global identity threshold by Usearch. The taxonomic assignment of OTUs was performed by searching against the SILVA v.132 rRNA sequence database using the VSEARCH v. 2.14.1 algorithm^43^.

### Sequencing of metagenomic DNA and identification of *dsrABD* gene sequences from Tom3 sample

Metagenomic DNA was sequenced using the Illumina HiSeq2500 platform according to the manufacturer’s instructions (Illumina). The sequencing of a paired-end (2 × 150 bp) TruSeq DNA library generated 108,273,070 read pairs.

Open reading frames (ORFs) with a minimum length of 96 nucleotides were predicted in all Illumina reads using OrfM v.0.7.1^44^. HMM profiles for *dsrA* and *dsrB*from TIGRFAM^45^, and for *dsrD* from Pfam^46^ were searched against all predicted ORFs using hmm search v.3.1b2^47^ with 1e^−3^ E-value cutoff. ORFs that had significant identity to the HMM profiles were further searched against Uniref100 database using diamond v.0.9^48^ with 1e^−3^ E-value cutoff. The NCBI taxonomy for every ORF homolog from Uniref100 database was identified using NCBI-taxonomist v.1.2.1 program (https://pypi.org/project/ncbi-taxonomist/).

### Enrichment and isolation of sulfate-reducing bacteria

The initial enrichments were set up in freshwater Widdel and Bak (WB) medium^49^ that contained (per liter) 4 g Na_2_SO_4_, 0.2 g KH_2_PO_4_, 0.25 g NH_4_Cl, 1 g NaCl, 0.4 g MgCl_2_·6H_2_O, 0.5 g KCl, 0.113 g CaCl_2_, 2 mL of vitamin solution, 1 mL of microelement solution, 1 mL each of Na_2_SeO_3_ (final concentration 23.6 µM), and Na_2_WO_4_ (24.2 µM) solutions, and solidified with 1.5% agar. Medium was adjusted to pH 7.2 with NaHCO_3,_ and Na_2_S·9H_2_O (0.36 g/L of Na_2_S·9H_2_O per litre of WB basal medium) was used as a reducing agent. Each cultivation vial received an iron wire (100% Fe) as previously described^40,50^. Formate (7.5 mM) was used as an electron donor for the enrichments. Culture vials (12 mL) were filled to the top, closed, and sealed by aluminium caps. The enrichment cultures were incubated at 28 °C. Black colonies, indicating a potential sulfidogenic growth, were selected and transferred to the liquid WB medium of the same composition. Pure cultures were isolated by repeated serial dilutions. The 16S rRNA genes were amplified using the primer pair 27F and 1492R^51^ and sequenced commercially by Syntol Co. (Moscow, Russia) using the Sanger method.

### Sequencing of *Desulfovibrio desulfuricans* L4 genome

Genomic DNA was extracted from strain L4 cells using a Power Soil DNA Isolation Kit (MO BIO Laboratories, Carlsbad, CA). The library for Illumina sequencing was prepared using the TruSeq Nano DNA Library Prep Kit (New England Biolabs, Ipswich, MA). Sequencing on the Illumina MiSeq generated 4,202,750 paired-end reads (2 × 300 nt, ~ 2 Gbp in total). Overlapping paired-end reads were merged using FLASH v1.2.11^41^, and low-quality bases were trimmed using Sickle v.1.33 (https://github.com/najoshi/sickle).

Genomic DNA of strain L4 was additionally sequenced on the MinION (Oxford Nanopore, Oxford, UK) using a Ligation Sequencing Kit 1D protocol and an R9.4 flow cell (FLO-MIN106). Sequencing resulted in 69,108 reads with a total length of ~ 348 Mbp. Hybrid assembly of Illumina and Nanopore reads was performed using Unicycler v. 0.4.8^52^. Two circular contigs of 3,544,025 bp and 10,876 bp, representing a chromosome and a plasmid were obtained. Gene search and annotation were performed using the RAST server 2.0^53^.

## Data Availability

The raw data generated from 16S rRNA gene sequencing and metagenome sequencing have been deposited in the NCBI Sequence Read Archive (SRA) and are available under the accession numbers SRR13442334–SRR13442345 and SRR13442943, respectively (BioProject PRJNA691721).The annotated genome sequences of *Desulfovibrio* sp. L4 have been deposited in the GenBank database under the accession numbers CP072608 (chromosome) and CP072609 (plasmid).
